# Avoiding culture shock with the SARS-CoV-2 spike protein

**DOI:** 10.7554/eLife.69496

**Published:** 2021-05-18

**Authors:** Benjamin G Hale

**Affiliations:** Institute of Medical Virology, University of ZurichZurichSwitzerland

**Keywords:** COVID-19, SARS-CoV-2, cell culture adaptation, furin cleavage site, serine proteases, airway organoids, Virus

## Abstract

When culturing SARS-CoV-2 in the laboratory it is vital to avoid deletions in the gene for the spike protein that could affect the interpretation of experiments.

**Related research article** Lamers MM, Mykytyn AZ, Breugem TI, Wang Y, Wu DC, Riesebosch S, van den Doel PB, Schipper D, Bestebroer T, Wu NC, Haagmans BL. 2021. Human airway cells prevent SARS-CoV-2 multibasic cleavage site cell culture adaptation. *eLife* **10**:e66815. doi: 10.7554/eLife.66815

Researchers across the globe are frantically trying to understand the biology of different SARS-CoV-2 variants to develop new therapeutics and bring the COVID-19 pandemic to an end. The process begins in the laboratory, with painstaking experiments to judge whether a drug can stop SARS-CoV-2 from replicating in cells in a dish, or whether a vaccine can stop an animal from getting sick. But such experiments require copious amounts of SARS-CoV-2, and the authenticity of that virus stock (which is usually cultured in animal cell lines) is paramount to ensure the validity of test results.

The standard 'go-to' cells for producing stocks of SARS-CoV-2 are derived from the Vero lineage (which are kidney cells isolated from an African green monkey almost 60 years ago). These cells are highly susceptible to viruses due to the absence of type I interferon cytokines, an important group of signaling proteins released by cells in the presence of viruses. Vero cells are a popular choice for isolating and propagating many viruses because they are well characterized and easy to maintain, they adhere to laboratory dishes, and they show visible structural changes when infected. However, like viruses circulating naturally in human populations, viruses grown in the laboratory also have a tendency to change and adapt to the environment that they are in.

An early study, since replicated by others, found that SARS-CoV-2 stocks cultured in Vero-derived cells often harbor mutations or deletions in the spike gene (Lau et al., 2020). The deletions remove an important region on the spike protein, called the multibasic cleavage site, which affects the ability of the virus to infect human airway cells (Hoffmann et al., 2020; Mykytyn et al., 2021; Pohl et al., 2021). Such viruses therefore do not behave like authentic SARS-CoV-2 in several aspects: they are less pathogenic (Johnson et al., 2021; Lau et al., 2020); they do not transmit (Peacock et al., 2021); and they exhibit altered sensitivities to inhibition by antiviral interferon-stimulated genes and patient antibodies (Johnson et al., 2021; Winstone et al., 2021).

These characteristics can make it difficult to interpret laboratory experiments that use Vero-produced viruses to assess the effectiveness of experimental drugs or vaccines. The ability of inactivated SARS-CoV-2 vaccines made in Vero cells to stimulate correct antibody responses might also be partially compromised, although this has not been formally assessed (Gao et al., 2020; Wang et al., 2020). Now, in eLife, Bart Haagmans and colleagues of the Erasmus Medical Center in Rotterdam and the University of Illinois at Urbana-Champaign – including Mart Lamers as first author – report simple methods for producing SARS-CoV-2 stocks in human cells that prevent such mutations and deletions in the gene for the spike protein (Figure 1; Lamers et al., 2021).

**Figure 1.**
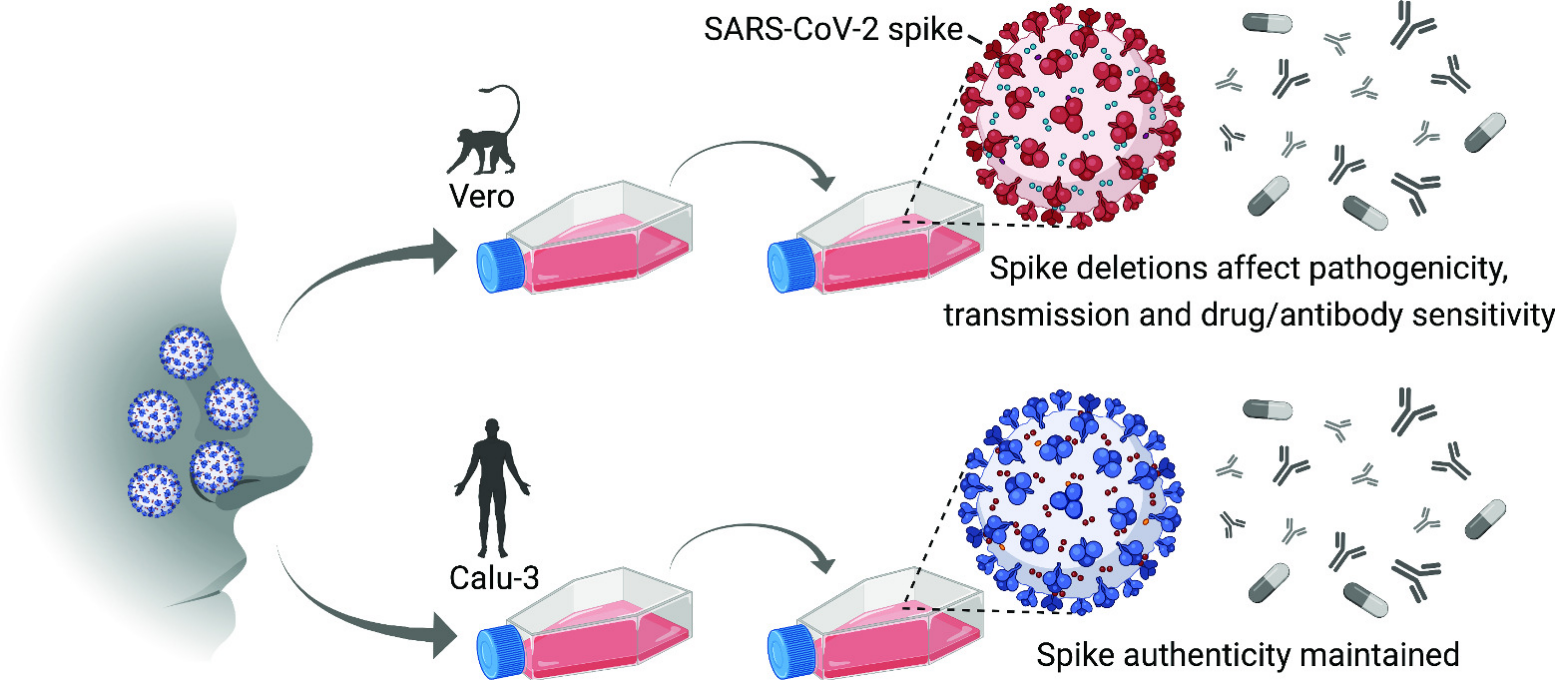
Culturing SARS-CoV-2 in Vero cells and human cells. Schematic representation of a human SARS-CoV-2 patient sample passaged using the Vero cell line (which was isolated from an African green monkey almost 60 years ago; top), or the Calu-3 cell line (which is a human cell line; bottom). Vero cells are deficient in a serine protease called TMPRSS2 that is needed for the virus to enter cells at the plasma membrane; however, certain SARS-CoV-2 variants with spike deletions (shown in red) can enter Vero cells via a different pathway, so these variants are artificially selected for and come to dominate the virus population. The pathogenicity, transmission properties, and sensitivity to antiviral drugs and antibodies of the variants are different to those of the wild-type virus. Calu-3 cells are not deficient in TMPRSS2, so the authenticity of the spike gene is maintained, and studies with such virus stocks more faithfully recapitulate human virus biology.

Figure created with BioRender.com.

First, Lamers et al. used a method called deep sequencing to confirm that repeated culture of SARS-CoV-2 in Vero cells leads to an increase in viral genomes with mutations or deletions in the important region of the spike gene. Less sensitive sequencing methods, or a reliance on 'consensus sequences', can leave researchers with the false impression that such deletions are absent.

Lamers et al. then pinpointed how these spike deletions give certain SARS-CoV-2 variants a replicative advantage in Vero cells, which enables them to dominate the virus population. Vero cells are deficient in a serine protease that is needed for SARS-CoV-2 to enter human airway cells at the plasma membrane: however, SARS-CoV-2 variants with the spike gene deletions exploit a different pathway (called endocytosis) to enter Vero cells. It seems the ability of the variants to take advantage of this second entry route allows them to dominate when Vero cells are used.

Lamers et al. next asked whether a human-airway cell line, Calu-3, might be a better alternative to culture SARS-CoV-2, given that this cell line possesses the necessary protease (Mykytyn et al., 2021). Indeed, they found that repeated culture of SARS-CoV-2 in Calu-3 cells (or Vero cells engineered to express the serine protease) prevented the accumulation of mutations and deletions in the SARS-CoV-2 spike. Moreover, Calu-3 cells were as good as Vero cells at producing the large amounts of virus necessary for further experiments. Thus, the authentic nature of SARS-CoV-2 stocks made in this way, as assessed by deep sequencing, will give confidence to the interpretation of subsequent experiments.

The results from this study make a compelling case for SARS-CoV-2 researchers to thoroughly characterize the genomic sequences of the virus stocks they produce (and to avoid consensus sequencing). Furthermore, researchers should consider the cells, the growth media and the additives used for virus production to prevent artefactual SARS-CoV-2 culture adaptations that might impact the evaluation of drug or vaccine effectiveness.

Cell culture systems to propagate viruses are the linchpin of virology, yet they have not really changed since the beginnings of the field. The application of modern technological advances, such as rational gene editing of cells or the use of *in vivo*-like organoid tissue models, promises to transform this critical aspect of virology and should allow researchers to update their methods to maintain experimental authenticity.
